# Bibliometric analysis of research on regenerative periodontal surgery during the last 30 years

**DOI:** 10.4317/jced.50646

**Published:** 2012-04-01

**Authors:** María M. Gutiérrez-Vela, Ana Díaz-Haro, Sonia Berbel-Salvador, Aldo Lucero-Sánchez, Nicolás Robinson-García, Antonio Cutando-Soriano

**Affiliations:** 1 Universidad de Almería; 2 Departamento de Biblioteconomía y Documentación, Universidad de Granada; 3 Departamento de Estomatología, Universidad de Granada

## Abstract

Objectics: The evolution of research activity during the last thirty years on regenerative periodontal surgery is studied. Results: A small number of authors are highly productive with more than 10 publications on the subject each. 79,6% of authors have only produced one article on the subject. The co-authorship average is of 2,68 authors per paper, with a collaboration between 2 and 6 authors. Main journals on the field of regenerative periodontal surgery are Journal of Periodontology and Journal of Clinical Periodontology, which are ranked 14th and 1st in their category according to the Journal Citations Reports. The most used language is English, followed by Japanese and Italian, Spanish occupying the eighth position. Conclusions: A significant increase on scientific literature is observed, similar to the one Dentistry has had. A reduced number of authors account for most production. In the same token, there is a scarce professionalization of researchers in this field, where most of the authors are occasional. On the other hand, there are two very specialized journals on this topic.

** Key words:**Bibliometrics, scientometrics periodontal regeneration, surgical periodontal treatment, scientific literature, scopus, scientific output.

## Introduction

The domain “Science of Science” came into existence in the 1960s through the convergence of Scientific Documentation, History of Science and Philosophy of Science. Its objective is to study scientific activity as a social phenomenon, using mathematical indicators and models (1). Bibliometrics can be defined as the science that studies the quantitative data derived from scientific publications (2). It studies the empirical evidence of scientific activity generated by authors and groups of collaborators through the final product of their research: the scientific article (3). Since the 1960s, numerous authors have revised this definition, highlighting the importance of studying the volume of scientific production, its growth, and the social structure of the groups who produce and use it—whether in Dentistry or in other disciplines (4,5).

Bibliometrics has grown and established itself as the fundamental methodological approach to the evaluation of scientific production and the phenomena linked to the communication of Science. As a discipline, it has two major areas of development and application (6). Firstly, through quantitative analysis of the scientific literature, we evaluate the development of Science and analyze the evolution of scientific production. Secondly, we analyze the editorial quality of scientific journals and their impact on the scientific community. This means that the results obtained and conclusions reached can help those responsible for any given journal to improve their management of that journal by, for example, facilitating decision-making on issues such as the selection of articles (7). Bibliometric techniques construct indicators that quantify the number of documents published by a country, research team or individual researcher, as well as the citations received by these documents (8). Bibliometric studies efficiently complement expert opinions and judgments within any given discipline and provide useful, objective tools that study the evolutionary processes at work in the results of scientific activity (3).

The objective of the present study is to analyze the development of scientific literature related to Regenerative Periodontal Surgery (RPS) during the period 1980-2010. Regenerative Periodontal Therapy (RPT) aims to restore the dental support apparatus lost because of trauma or periodontal illness (9). In the field of Periodontology, it has been the focus of much recent research. The recovery of periodontal tissue lost as a consequence of illness remains one of the clinician’s most sought-after ideals when treating patients (10,11).

Regenerative Periodontal Therapy has developed spectacularly in both scientific and technological terms. Some of the regenerative procedures available can obtain results that we could term Periodontal Regeneration (12,13) which is defined, therefore, as any procedure or technique entailing the formation of new periodontal ligament, new acellular cement and new bone with histologically-evaluated, inserted, connective fibers. The procedures that have produced true periodontal regeneration are autogenous bone grafting, guided tissue regeneration (12) and enamel matrix proteins (EMD) (14).

To date, no study has analyzed scientific production in RPT. However, within Dentistry, studies of other fields—e.g. Pediatric dentistry (15), Orthodontics (16)—have been published. Yang et al. analyzed the dental literature in seven subdisciplines of Pediatric dentistry (dental implants, endodontrics, oral radiology, oral surgery, orthodontics, periodontology and restorative dentistry) between 1989 and 1998. Following a bibliometric analysis of MEDLINE, they compared results for these subdisciplines in adolescents and in adults. They concluded that pediatric dentistry is represented by a considerable volume of literature that facilitates clinical decision-making, that the subdisciplines vary significantly, and that the volume of the literature is increasing yearly (15).

In a bibliometric analysis of the field of orthodontics (16) that also used MEDLINE, Mavropoulos et al. revie-wed the literature published in the most important journals in Dentistry and Orthodontics between 1981 and 2000. They concluded that the number of English-language articles on Orthodontics had increased during the period and that almost half (45%) were published in specific journals. The results of both of these studies coin-cide with those obtained in the present bibliometric analysis of RPS.

Similar studies have been conducted in disciplines such as Psychology (17,18), Medicine (19,20) or Education (21,22).

The objectives of the present study are: to apply bibliometric methodology to articles on RPS and thus attempt to demonstrate that the substantial developments in the field have been reflected in the scientific documentation generated in the period 1980-2010; to examine the distribution of publications in terms of document type-research articles, review articles, articles in press, or conference proceedings in paper format; to analyze document production by author and determine the level of inter-author collaboration; to determine which language is most used in publications in this field; and to identify the most productive journals in RPT over the three decades.

## Material and Methods

We conducted an exploratory analysis of the scientific literature on RPS.

To locate all the documents, we used the Scopus database (Elsevier). Scopus covers a wider spectrum of journals than other databases also used for searches in Dentistry, such as PubMed and Web of Science (WoS) (8). It is larger than WoS and represents 50% of the universe of journals, whereas WoS represents only 25%. Scopus records over 5300 serial publications in the medical sciences. It includes more journals in Spanish and in other European languages than WoS; hence it has fewer language and geographic biases. All PubMed-indexed journals are processed in Scopus too. However, as it is a multidisciplinary database, Scopus also covers topic-related publications that are not strictly considered medical.

The search was limited to the period 1980-2010 because we consider 30 years to be a long enough period to demonstrate the extent to which both Dentistry in general, and RPS in particular, have advanced. We began with an initial search of the Medical Subject Headings (MeSH) in the PubMed database. In Medicine and the Health Sciences, MeSH is the most widely used listing of discipline headings. It has a hierarchical structure and is regularly updated by the US National Library of Medicine to introduce new concepts as they appear in the literature. ( Table 1,  Table 2)

**Table 1:**
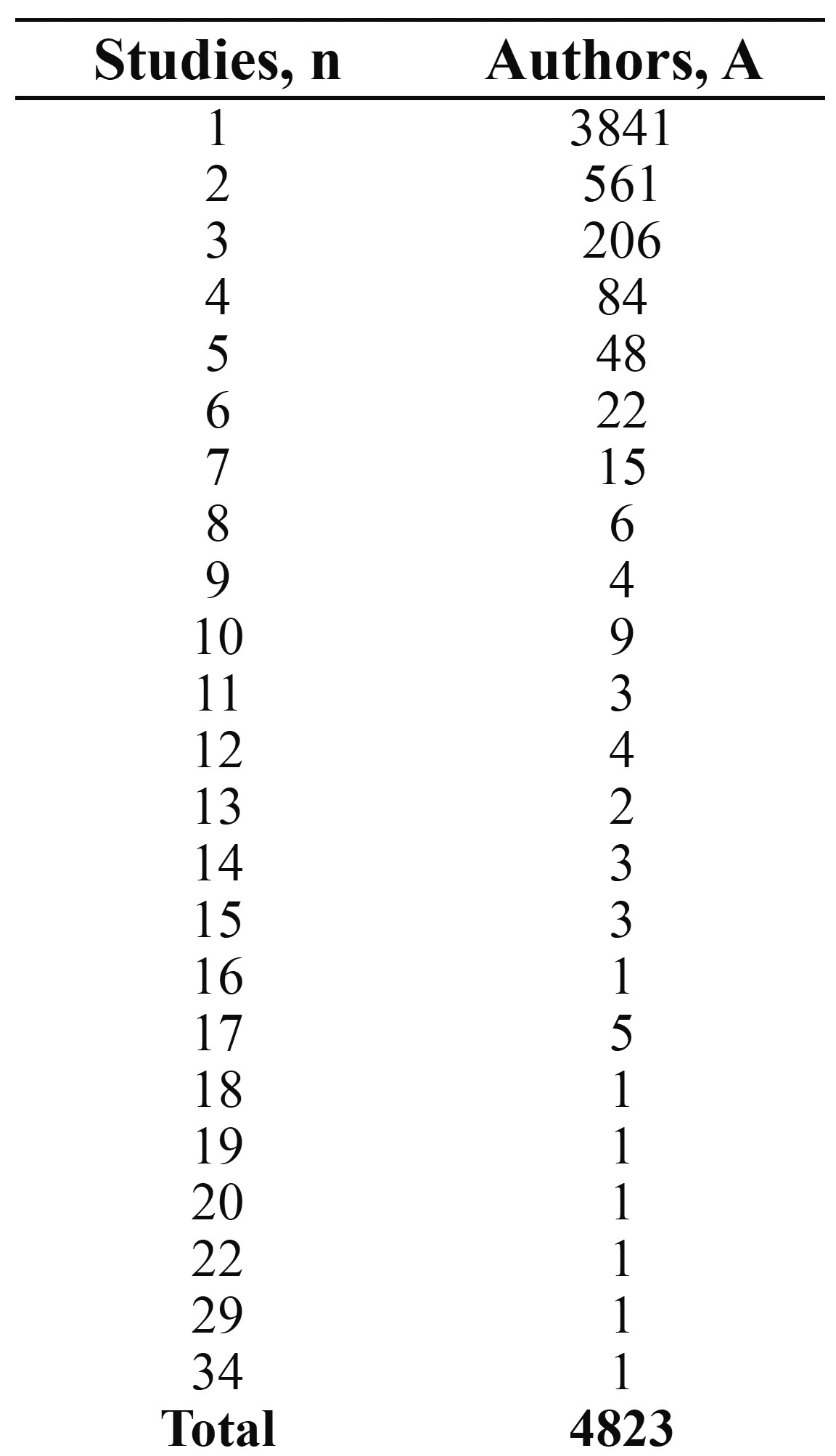
Distribution of the number of authors by the number of studies published.

**Table 2:**
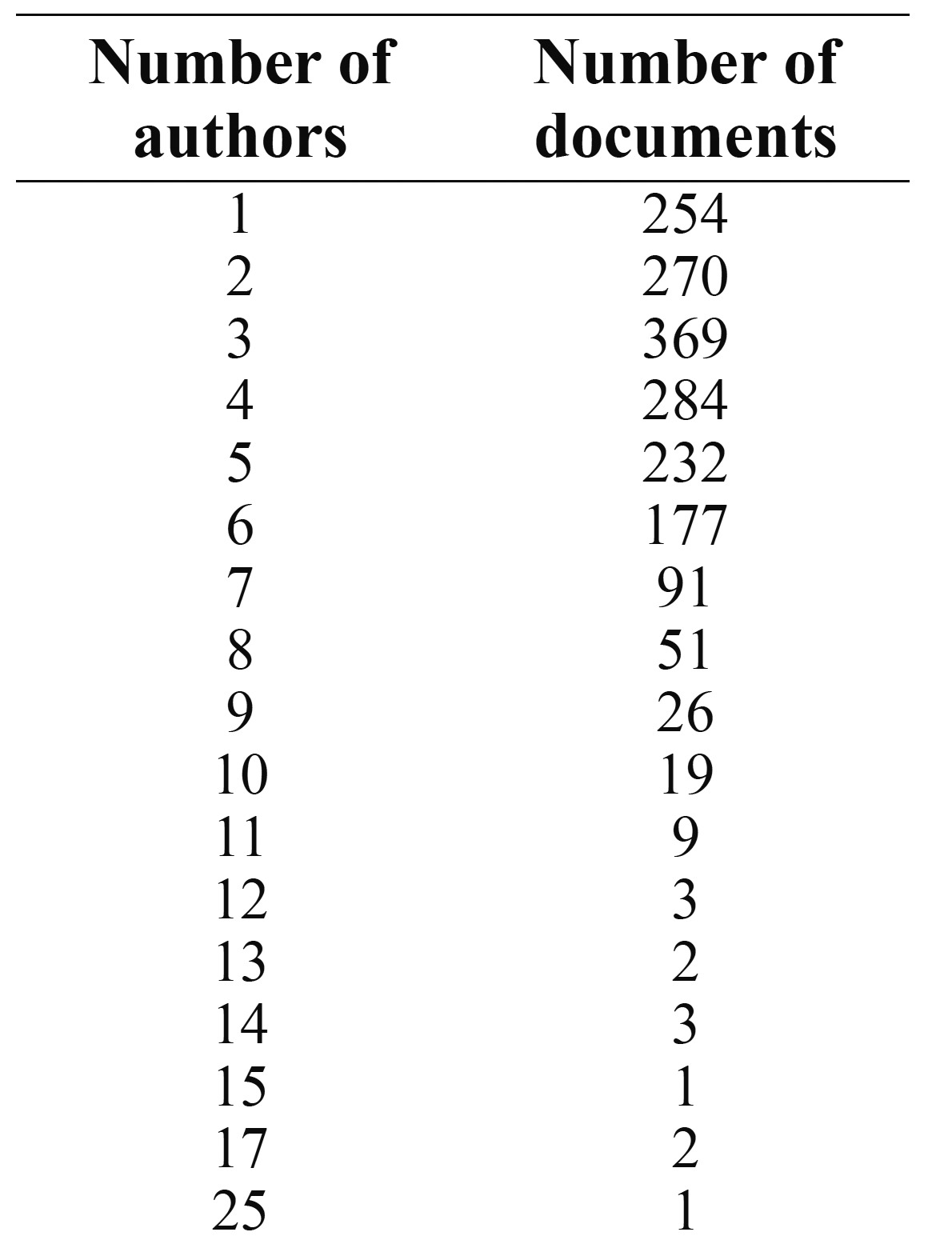
Distribution of the number of documents with n authors.

Precisely because of its validity and widespread use by the scientific community, we considered it essential to use MeSH to identify the descriptors and their synonyms that would enable us to identify the topic being studied with maximum precision. Having extracted the appropriate terms, these were translated into the Scopus database’s search language, which is based on free text and Boolean logic. This process was conducted under the supervision of subject matter experts. The equation finally used was:

“periodontal regeneration” OR “surgical periodontal treatment” OR “periodontal surgery”.

The data obtained were exported to a Microsoft Excel spreadsheet and reviewed manually.

We first conducted a quick analysis of the evolution of the scientific production of documents related with the topic being studied. We contrasted this with the overall evolution of Dentistry to establish the subdiscipline’s growth pattern.

To study productivity by author, we calculated the Lotka productivity index. This is calculated from a decimal logarithm of the number of publications, enabling us to group authors in levels of productivity. These are normally banded into three groups: small producers, with only one publication and an index of 0; medium-sized producers (2-9 publications), with a Lotka productivity index score of 0-1; and large-scale producers (≥ 10 publications), with a productiviy index of ≥ 1. Lotka’s law describes the quantitative relation between the number of authors and the articles published over a specific period of time, distributing them according to their productivity (6,23). It demonstrates that the production of publications is distributed asymmetrically as most documents are published by a small proportion of the most productive authors (24). The relation between the collaboration index and the Lotka index should result in a positive correlation.

We also calculated the coauthorship index, which is obtained by dividing the total number of signatories between the total number of documents or studies on the topic being studied:

Coauthorship index = Total number of signatories / Total number of documents.

And the rate of single authorship:

Rate of single authorship = Number of documents by a single author / Total number of documents.

Finally, we analyzed the type of journal which publish studies on this topic, identified the most productive journals, and analyzed their position in the Thomson Reuters *Journal Citation Reports* (JCR) database, according to their respective impact factors. Thus, we were able to determine how much one database overlapped with the other, and—what proved more interesting—determine the impact of the journals that publish articles on RPS.

## Results

1. Analysis of production

1.1. Number of publications per year

Between 1980 and 2010, 1794 documents were published on RPS. Productivity was at its highest in 2008.

We found exponential growth, with 18.6% of documents published in the first decade, 26.7% in the second, and 54.7% in the third. The second decade of the study period saw slight growth by comparison with the first; the third decade saw highly substantial growth which almost doubled that of the second.

**Figure 1:**
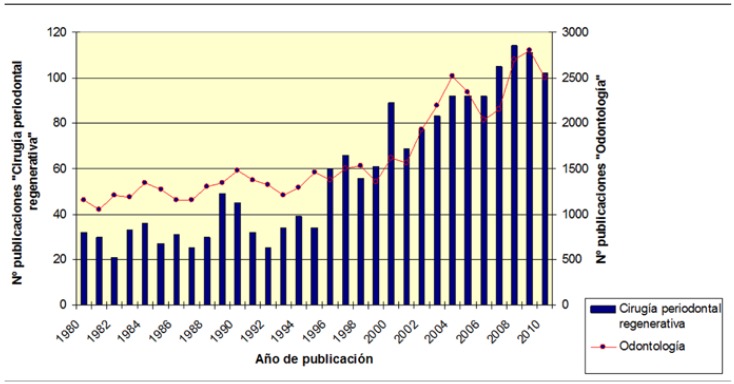
Distribution of the number of documents on Regenerative Periodontal Surgery and Dentistry, published per year.

**Figure 2:**
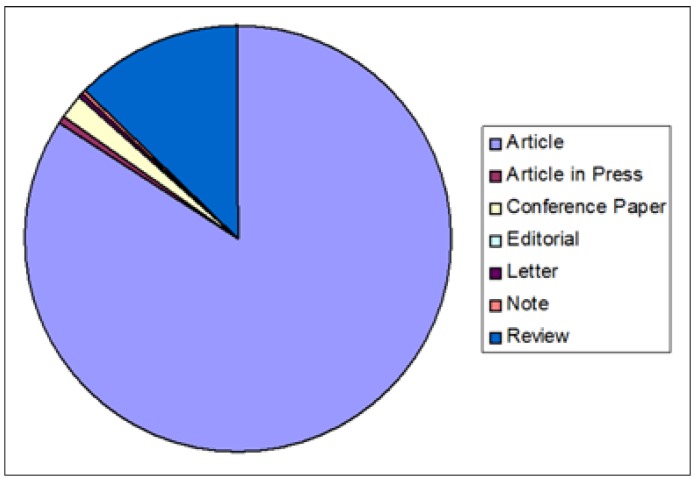
Distribution of publications by document type.

The increase in production was especially significant after 1996 (Fig. 1,2). We found that the increased research and the growing number of publications in RPS followed a pattern similar to that recorded in the other subdisciplines within Dentistry.

In Dentistry as a whole, the first decade of the study period accounted for 20.2% of the documents; the second, 43.3%; and the third, 36.3%. The greatest increase occurred in the second decade; in the third decade producti-vity fell, most notably between 2005 and 2007.

1.2 Distribution of publications by document type.

Most documents (84.11%) were research articles; 12.76% were review articles; and the remaining 3.12% were categorized as articles in press, conference proceedings in paper format, editorials, letters or notes.

2. Analysis of production

2.1 The lotka productivity index

Some 4823 authors had published on RPS but only 36 (0.7%) were large-scale producers with at least 10 publications. In all, 79.6% of authors had published just one article.

We found that for documents on RPS, Lotka’s Law was fulfilled. Some 0.7% of authors accounted for 76.9% of the documents produced.

**Figure 3:**
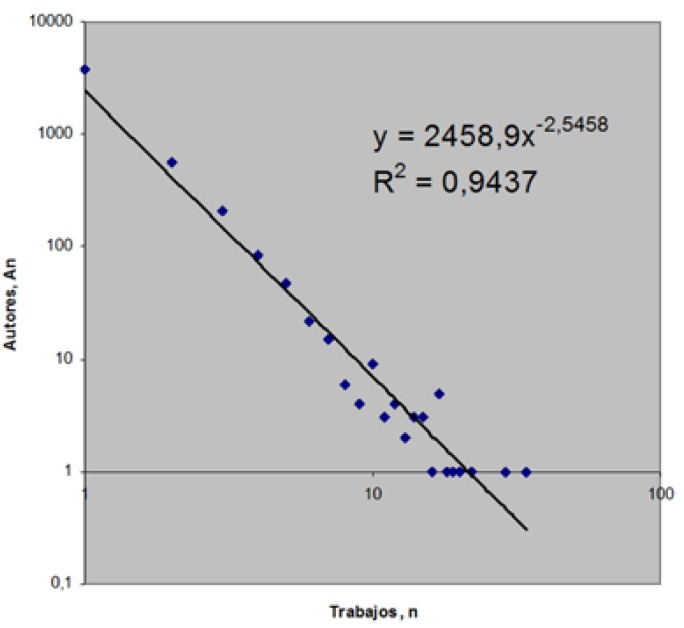
The Lotka productivity index.

The Lotka productivity index is shown in Fig. 3.

Among the authors who had published on RPS, the following stand out: A. Sculean with 55 documents, U.M.E. Wikesjo with 34, and D.L. Cochram, with 29.

2.4. Coauthorship index, or signatories per study.

In Science, collaboration between authors is an important means of advancing knowledge. At times, multidisciplinary challenges must be faced and scientists have to collaborate with each other (25).

In the present study, the coauthorship index was 2.688, revealing a high level of collaboration between authors in the field of RPS.

2.5. Rate of single authors

The single author rate was 0.142 with 14.2% of documents signed by a single author.

2.6 Distribution of the documents and authors

As we have said, 14.2% of the documents were the product of a single author, whereas 85.8% were signed by more than one author.

Collaboration between 3 authors represented 20% of documents and was the most frequent.

Some 11% of the total were signed by more than seven authors and only 1% by more than 11.

The most frequent level of collaboration was that between 2 and 6 authors, at 72.24%.

The mode was 3.

3. Most productive journals

The ten most productive journals on this topic and their respective positions in the JCR appear in  Table 3.

**Table 3:**
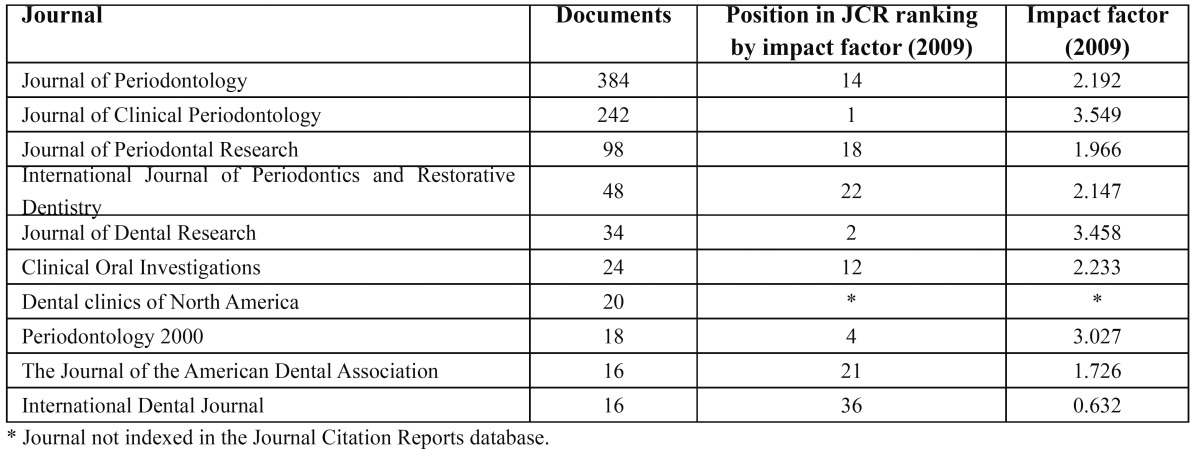
Most productive journals.

During the study period, 411 journals published documents on RPS.

Among these, 2 stand out: the *Journal of Periodontology*, with 384 documents, and the *Journal of Clinical Periodontology*, with 242. These two journals account for 34.8% of all publications on RPS.

We found that the four most productive journals—these two, together with the *Journal of Periodontal Research*, with 98 documents, and the *International Journal of Periodontics and Restorative Dentistry*, with 48—account for 43% of all publications. These four journals are the most productive.

During the study period, 94.6% of journals published fewer than ten articles on RPS.

4. Language of publication

Some 86.3 % of the documents found were in English; the second most frequently used language was Japanese with 2.51%; and the third was Italian, with 2.45%; Spanish was eighth, with 0.50%. ( Table 4)

**Table 4:**
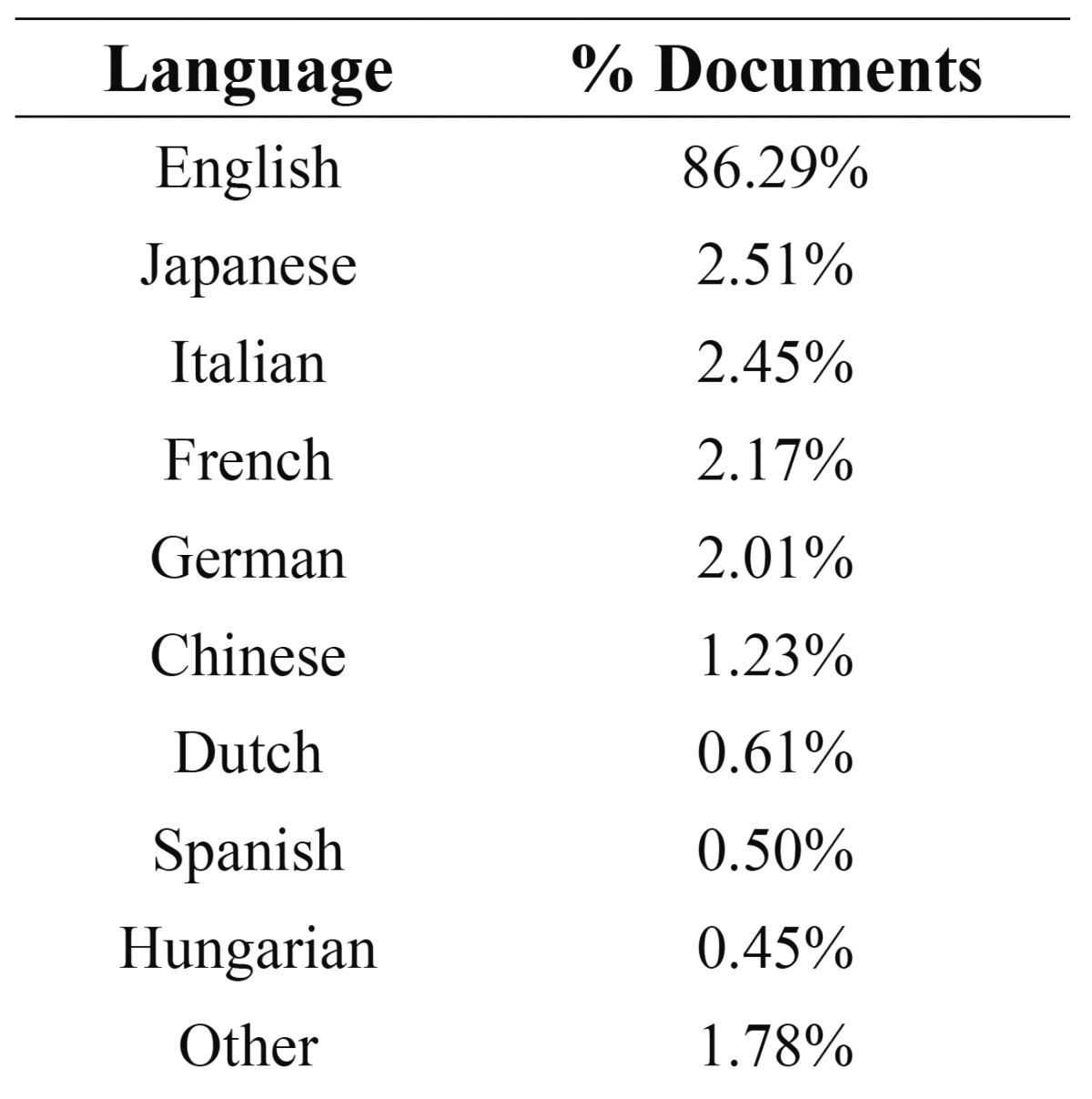
Distribution of documents by language of publication.

## Discussion

The increase we found in scientific production on RPS is due to the overall growth in scientific production worldwide (26-29) and not to any specific demand for this topic.

If we divide the study period into 3 decades, we find that growth has been greater since 1996 and, above all, during the last decade, when the number of publications doubled by comparison with the previous decade. In Spain, this may be due to the creation of University Faculties of Dentistry in 1996, when Dentistry ceased to be considered a subspecialty of Medicine. This led to substantial growth in Dentistry and a greater degree of specialization; an increase in the number of professional researchers; the need for teachers in the new Universities and a consolidation of the academic status of the discipline. It would, however, be of interest to analyze the scientific literature published in Spain that might support this view. Notwithstanding, the field has developed considerably in recent years. Principally, due to the appearance of new techniques and materials which can be applied in RPS (14,30).

A very low percentage of authors account for more than a third of the publications because only a small proportion of RPS professionals dedicate themselves to research on a long-term basis such that they can produce a significant number of documents.

The index of transitoriness in this field is 79.6% which shows that RPS is not yet a soundly established discipline and lacks a firm base of dedicated researchers. Perhaps this is because RPS is primarily of clinical application and, therefore, most RPS professionals do not undertake research. We can only hope that the index of transitoriness will fall.

The mean number of authors per article was 2.68 (range 2-6), and the mode 3.

Only 14.2% of publications are single-author documents and the remaining 85.8% have two or more authors. These data on author collaboration are important indicators that reflect the importance of teamwork and the increasing professionalism of the scientific community in Dentistry. As Science and Dentistry have evolved, research has become more complex and more specific and researchers need to collaborate with other research teams to conduct their studies (28,6).

The four most productive journals account for most of the documents. Among these, the *Journal of Periodontology* and *the Journal of Clinical Periodontology* stand out. Regenerative Periodontal Surgery constitutes a highly specific field within Periodontology and the fact that specific journals do specialize in the field enhances its importance. The impact factor measures the importance of highly influential scientific publications. The JCR for 2009 included in the category for Dentistry, nine of the ten most productive journals identified by our study. The second most productive journal, *the Journal of Clinical Periodontology*, led the category with an impact factor of 3.549; the first most productive journal, *the Journal of Periodontology* was ranked 14th out of 64. Bearing this in mind, we conclude that Periodontology and, therefore, RPS has a high level of impact on research in Dentistry.

As in all fields of Science, English is the most frequently used language of publication as it is clearly the *lingua franca* of the scientific community (31,32)

## Conclusions

The growth of scientific production in RPS follows the same pattern as that of scientific production around the world. In recent years, substantial growth has been observed, which is due to the methodological and technical advances and the development of new materials in the discipline, and to the establishment of Dentistry as a sound discipline within university curricula in the 1990s. The distribution of document authorship fulfills Lot-ka’s law, which states that a small core of authors disseminates knowledge to the greater part of the scientific community. Research is conducted by teams, which are the fruit of the collaboration needed to conduct studies like these that require the participation of different institutions with different profiles. The principle journals that publish on RPS have high impact factors, which reflects its current relevance to the scientific community. English is the *lingua franca* of Science and of this particular subdiscipline. Spanish ranks eighth among languages of publication.
